# A cognitive behavioural group treatment for somatic symptom disorder: a pilot study

**DOI:** 10.1186/s12888-023-05141-9

**Published:** 2023-11-30

**Authors:** Katherine Jongsma, Bri Susanna Darboh, Sasha Davis, Emily MacKillop

**Affiliations:** 1 Department of Psychiatry & Behavioural Neurosciences, McMaster University, Hamilton, ON Canada; 2 Clinical Neuropsychology Service, St. Joseph’s Healthcare Hamilton, Hamilton, ON Canada; 3 Department of Psychology, York University, Toronto, ON Canada

**Keywords:** Somatic symptom disorder, Somatization, Somatoform disorder, Medically unexplained symptoms, Pain, Group cognitive behavioural therapy, Healthcare utilization, Interventions

## Abstract

**Background:**

Somatic symptom disorder (SSD) presents challenges to the healthcare system, including frequent medical visits, lack of symptom relief experienced by individuals with this condition, high associated medical costs, and patient dissatisfaction. This study examined the utility of a novel, low-barrier, brief cognitive behavioural therapy (CBT) group intervention for individuals with SSD.

**Methods:**

Participants were referred by their mental health providers or self-referral. Each participant underwent a telephone screen and in-person psychological and neuropsychological screen. Two cycles of the CBT-based group (*n* = 30), each consisting of six weekly two-hour sessions, were facilitated at a large outpatient mental healthcare facility in Ontario, Canada. The final sample consisted of 13 individuals of whom 11 completed the treatment. Clinical outcome measures were administered pre-, mid- and post-group, including the Generalized Anxiety Disorder–7, Perceived Stress Scale–4, Pain Self-Efficacy Questionnaire, Pain Disability Index, Revised Illness Perception Questionnaire, and sections of the Patient Health Questionnaire. Six healthcare utilization metrics were collected from electronic medical records at six months pre- and post-group. Paired samples t-tests were used to examine pre- to post-group differences in participants’ somatic symptoms, psychological functioning, health, and degree of healthcare utilization.

**Results:**

When comparing pre- and post- group, we observed reductions in the mean scores for somatic symptom severity, depressive symptomatology, anxiety, perceived stress, and perceived disability related to pain. The change in depressive symptomatology yielded a small effect size (*d* = 0.30). Further, we observed downward trends across participants’ pre- to post-group healthcare utilization, with small effect sizes observed for hospital admission (*d* = 0.36), days admitted to hospital (*d* = 0.47), and inpatient consults (*d* = 0.42). Differences between pre- and post-group measures of somatic symptom severity, psychological functioning, health, or healthcare utilization did not reach significance.

**Conclusions:**

Current findings provide support for the potential effectiveness of an abbreviated CBT group for individuals with SSD in reducing psychiatric symptomatology. Further research is recommended, including randomized control trials, cost-benefit analyses, and comparisons between abbreviated versus longer-duration treatment programs for SSD.

**Trial registration:**

Not applicable.

## Background

Somatic symptom disorder (SSD) is characterized by prominent physical symptoms that are associated with marked distress and impairment, including excessive thoughts, feelings, and behaviours relating to the physical disturbances [1]. It is well-documented that the ongoing distress about physical symptoms experienced in SSD promotes a self-perpetuating feedback loop between increased somatic and exacerbated psychological symptoms. Western biomedical perspectives propose that the development of SSD is facilitated by an individual’s heightened awareness of bodily sensations and a concurrent propensity for interpreting these sensations as secondary to medical illness [2]. Ontario has a diverse and multicultural population; thus, it is imperative to consider multicultural perspectives of somatic symptoms. The current study was conducted in the City of Hamilton, which is situated on the traditional and ancestral territories of the Erie, Neutral, Huron-Wendat, Haudenosaunee, and Mississaugas. Many Indigenous cultures emphasize the notion of balance across the mental, physical, spiritual, and emotional aspects of one’s life, within the social and community context [3–7]. This holistic model of health and wellbeing is often represented through the concept of the medicine wheel. With respect to cultural idioms of distress, many cultures (including as Asian and Indigenous cultures) are more likely to express their distress through physical symptoms instead of psychological complaints, and some cultures have specific presentations of mental disorders with somatic components such as ‘hwabyung’ in Korea, ‘shenjing shuairuo’ in China, and ‘brain fag’ in Nigeria [8]. Applying Western diagnostic criteria for somatic disorders to those in different cultures runs significant risk of lower diagnostic validity and over-pathologizing. The DSM-5 expanded the diagnostic criteria of SSD to include symptoms associated with a known medical condition, in addition to symptoms which are medically unexplained [1]. With the changes in diagnostic criteria, prevalence estimates have risen to comprise between 5 and 7% of the general population [1]. Individuals with SSD present to many medical specialties and in ambulatory care settings given the high comorbidity with a number of medical and psychiatric conditions, including: irritable bowel syndrome, chronic pain, major depressive disorder, generalized anxiety disorder, phobic disorders, posttraumatic stress disorder, borderline personality disorder, and history of child abuse [9–13]. Indeed, SSD demonstrates considerably high prevalence rates and co-occurrence with chronic medical and psychiatric conditions. Those with functional somatic syndromes such as SSD face perceived stigma [14, 15] as well as stigmatization from their healthcare providers [16–18] and the general public [19, 20], which can serve as a barrier to treatment. These issues indicate a crucial need for healthcare systems to more effectively treat and manage SSD.

SSD is a serious and debilitating disorder with wide-ranging adverse biopsychosocial underpinnings and implications. High levels of healthcare utilization are characteristic of this patient population and the frequency of these behaviours in SSD has been associated with adverse outcomes. Specifically, high healthcare utilization in SSD often leaves individuals vulnerable to incurring insurmountable healthcare expenses, as well as time spent seeking treatment alternatives that far exceed national averages in the United States [21]. In fact, it has been reported that U.S. medical costs associated with somatization (i.e., production of somatic symptoms with no discernible cause or in excess of medical etiology) exceed $256 billion annually [22]. Due to the limited offerings for mainstream care, patients with SSD often independently seek unconventional and alternative treatments that are ultimately unsuccessful or minimally beneficial in mitigating symptoms, perpetuate health-seeking behaviors, and contribute to higher frustration and dissatisfaction with the healthcare system among these individuals [23]. In keeping with this pattern of inadequate access to care, long-term prognostic outcomes for SSD are poor, as evidenced by high rates of disability in a longitudinal study of SSD outcomes [24]. Indeed, rates of disability in SSD exceed levels observed in most other psychiatric disorders [25]. It is unclear whether this is a consequence of ineffective interventions or due to the difficult nature of the symptom presentation in SSD. Nonetheless, research indicates that practitioners frequently have difficulty implementing effective treatment approaches for SSD. Taken together, these challenges in providing effective SSD interventions, the high frequency of healthcare visits among SSD patients, the costly nature of the associated medical services, and the high rates of disability among individuals with SSD all contribute to the burgeoning global burden of disease and indirect costs to the economy (e.g., lost work productivity) [21, 26].

Despite the high prevalence rate of SSD and the robust correlation with adverse functional outcomes (i.e., reduced quality of life, high rates of healthcare utilization), the literature on interventions for the treatment and management of SSD remains underdeveloped. Consequently, adequate treatment for SSD continues to prove as a challenge for healthcare practitioners. While considerable strides have been made in attempting to develop evidence-based treatment approaches, recommendations, and protocols for the successful management of SSD, many of the interventions have significant limitations [23]. Although there is marginal support for the use of new-generation antidepressant medications in treating SSD [27], evidence for the effective pharmacological treatment of SSD is equivocal, and it is generally not a widely utilized intervention strategy. Still, medication is often used to treat co-occurring symptoms and conditions, which may promote the use of multiple medications and ultimately increase the risk of side effects from polypharmacy. The two interventions with the strongest empirical support are the psychiatric consultation intervention (PCI) and cognitive behavioural therapy (CBT). PCI involves advising the primary care physician to examine patients with SSD during regularly scheduled appointments, while limiting the use of alternative diagnostic and intervention approaches [28]. Studies have demonstrated that the implementation of the PCI into the treatment regimen of SSD patients has facilitated significant declines in subsequent hospitalization, unnecessary procedures, and healthcare expenditures [28].

Regarding psychotherapeutic intervention for the treatment of SSD, the bulk of research efforts have centered on exploring the utility of CBT and third wave CBT (e.g., mindfulness) interventions. Although there is emerging evidence to support the use of mindfulness therapy in SSD [29], this research is still in its infancy. According to meta-analyses and systematic reviews, at present, CBT is the only psychological treatment to have been studied thoroughly enough to begin to substantiate itself in the literature as a potentially useful approach for the management of SSD [30–32]. Specifically, studies have demonstrated efficacy of CBT interventions for SSD when compared to the status quo of care or wait-listed patient groups [32, 33]. Individual and group CBT for SSD facilitated reductions in somatic symptom severity that typically outperform other treatment frameworks in clinical settings [32, 33]. Further, reduction in healthcare utilization has also been observed following individual CBT treatment for SSD [21]; however, effect sizes are typically small and vary widely across studies [32]. To date, studies examining CBT groups for somatic syndromes have been conducted in the United States, Spain, Denmark, Sweden, and the Netherlands, spanning 8–13 sessions [21, 34–37]; few of these have evaluated the impact of group CBT on healthcare utilization outcomes.

The current pilot study aimed to examine the utility of a brief six-session CBT group intervention focused on ameliorating somatic and comorbid psychiatric symptoms, and reducing healthcare utilization for individuals living with SSD in Ontario, Canada.

## Methods

### Study design

The current pilot study was an uncontrolled analysis of a CBT-based group treatment for SSD designed to examine symptom reduction in response to treatment and clinical feasibility. Two groups were facilitated between May and December of 2019. The study was conducted at a large outpatient mental healthcare facility in Ontario, Canada, and was approved by the institution’s human research ethics committee.

### Participants

There were 30 potential participants (28 clinician referrals, 2 self-referrals) across two cycles of the intervention group. Eighteen of these individuals consented to the research study and were eligible to participate in the group, six people declined, four were ineligible for the group because they were not followed by a clinician at the mental healthcare facility (a requirement for group participation), and two were unable to be contacted after they were referred. Of the 18 people who participated in the group, 12 completed at least four out of six sessions and were considered treatment completers (33.3% drop out rate). Five participants were not included in data analysis as their scores on the somatic symptom scale of the Personality Assessment Inventory (PAI) were lower than a *T* score of 70, indicating that they did not endorse clinically significant symptoms of SSD at the outset of the group. The final sample consisted of 13 individuals (73% female) ranging from 32 to 69 years old (*M* = 43.55, *SD* = 11.87), of whom 11 were treatment-completers (i.e., attended ≥ 4 sessions) and 92% (*n* = 12) had been previously diagnosed with SSD (see Table 1 for participant details).

Of the 11 treatment-completers, the average age was 43.55 (ranged from 33 to 69). Eight were women (See Table 1). Ethnically, ten were white and one was biracial. Two were employed full-time, one was retired, and the remaining were unemployed. Annual family income ranged from $0 to over $200,000, with 45% of participants making between $0–39,999. In terms of their level of education, two participants had a high school education, four had some college or university (no diploma or degree), two had college diplomas, two had university degrees, and one had a graduate degree.

**Table 1:** Sociodemographic characteristics of the somatic sample at baseline

	Completers (*n* = 11)		Non-Completers (*n* = 2)		Full Sample (*n* = 13)	
	Mean	SD	Mean	SD	Mean	SD
Age	43.55	11.87	36.50	3.54	42.46	11.20
Education	13.64	2.11	12.00	0	13.38	2.02
Males	*n* = 3		*n* = 1		*n* = 4	
Females	*n* = 8		*n* = 1		*n* = 9	

*Note.* Participants were included in the somatic sample if their Somatic Complaints Scale (SOM) Total score on the PAI exceeded a T score of 70

### Recruitment

Participants were recruited through referral from mental health providers at the hospital where the study was being conducted, as well as advertisements posted throughout the hospital. Eligibility for the treatment group was determined in a two-part screening process. First, potential participants were contacted by one of the group facilitators via phone and were provided with a detailed account of what participation in the intervention would entail. They were invited to take part in the research project but were still offered participation in the treatment group as part of their clinical care even if they declined to have their clinical data included in the research study. If potential participants indicated that they were interested in taking part in the research study, group facilitators conducted an initial structured screening to determine eligibility. If deemed eligible, a time was arranged for the participant to attend the clinic for the second part of the screening process.

The second stage of screening consisted of an individual appointment to complete the research study consent form, a demographic questionnaire, a questionnaire related to personality and psychological distress, and a cognitive assessment consisting of the Test of Premorbid Functioning [38], Wechsler Adult Intelligence Scale, Fourth Edition – Digit Span subtest [39], Trail Making Test [40], and Repeatable Battery for the Assessment of Neuropsychological Status Update [41] for the purpose of potentially analyzing the neurocognitive profile of SSD in a future study if the group is deemed feasible and continued to be offered. Individuals were eligible to participate based on the following inclusion criteria: (i) 18 years or older, (ii) formal diagnosis of SSD or functional neurological symptom disorder, (iii) deemed by one of the group facilitators to have clinically significant distress or functional impairment related to somatic symptoms during the phone screening, and (iv) being followed by a healthcare practitioner at the facility. Exclusion criteria included: (i) acute/severe suicidality, (ii) severe PTSD symptoms, (iii) current psychosis, (iv) severe substance use, and (v) cognitive impairment that would interfere with engagement in the group (i.e., intellectual disability, mild cognitive impairment, or dementia).

### Measures

#### Clinical measures

The measures were administered at pre-group (session 1), mid-group (session 3/4), and post-group (session 6) with the exception of the PAI, which was administered during the in-person screening appointment at baseline only.

**Personality Assessment Inventory (PAI).** The PAI [42] is a widely used 344-item self-report measure of personality and psychopathology. The PAI contains validity, clinical, interpersonal, and treatment consideration scales that can be useful in diagnosis and treatment planning. The indices have shown strong reliability and validity across community and clinical samples with Cronbach’s alpha values ranging from 0.81 to 0.86 [42, 43]. The anxiety, depression, and somatic symptom scales were included in the current analyses.

**Patient Health Questionnaire 9 (PHQ-9).** Depressive symptoms were measured with the PHQ-9 (Kroenke, Spitzer, & Williams, 2001), which is the 9-item depressive symptom module of the Patient Health Questionnaire (PHQ) [44]. It is a self-report tool that assesses current symptoms of depression during the past two weeks. Scores range from 0 to 27, with higher scores indicating higher levels of depression. Good internal consistency (Cronbach’s α = 0.86–0.89) and test-retest reliability (intraclass correlation = 0.94) have been reported [45].

**Generalized Anxiety Disorder 7 (GAD-7).** Anxiety-related symptoms were measured using the GAD-7 [46], a 7-item self-report scale that assesses symptoms of generalized anxiety disorder during the last two weeks. GAD-7 scores range from 0 to 21, with higher scores reflecting higher anxiety levels. Evidence has provided support for strong validity, excellent internal consistency (Cronbach’s α = 0.92), and good test-retest reliability (r = .83) [46].

**Patient Health Questionnaire 15 (PHQ-15).** Somatization was measured with the 15-item somatic symptom module of the PHQ [47], which assesses how much the respondent has been bothered by somatic symptoms in the past four weeks. PHQ-15 scores range from 0 to 30, with higher scores indicating greater somatic symptom severity. Previous studies have indicated good internal consistency (Cronbach’s α = 0.80), test-retest reliability (r = .60), and validity [47, 48].

**Perceived Stress Scale 4 (PSS-4).** Stress levels were measured with the 4-item PSS-4 [49], which measures self-reported stress levels in the past month. While the validity and reliability of this abbreviated version of the PSS tend to be weaker than the original measure due to the small number of items (Cronbach’s α = 0.60–0.82) [50], its brevity was important in the current study and its psychometric properties were deemed adequate.

**Pain Self-Efficacy Questionnaire (PSEQ).** The 10-item PSEQ [51] was used to evaluate the confidence people have in performing activities while in pain. Scores range from 0 to 60, with higher PSEQ scores being associated with better functional ability. The measure has demonstrated excellent internal consistency (Cronbach’s α = 0.92) [51].

**Pain Disability Index (PDI).** The 7-item PDI [52] was used to measure the extent to which pain interferes with engagement in activities. The total score ranges from 0 to 70, with higher scores reflecting higher inference of pain with daily activities. Studies have found the PDI to be reliable (Cronbach’s α = 0.85–0.86) and valid [53].

**Revised Illness Perception Questionnaire (IPQ-R).** The IPQ-R [54] is a 56-item questionnaire used to measure components of illness representation. Subscales include identity, consequences, timeline-acute/chronic, timeline cyclical, treatment control, personal control, illness coherence, emotional representation, and cause. The subscales have been found to have good internal consistency, test-retest reliability, and validity [55].

#### Healthcare utilization

Healthcare utilization was assessed by deriving data from the electronic medical records of healthcare contacts during the six months prior to and following participation in the group. Information accessed included six different types of utilization, including: (i) total number of outpatient appointments (in-person or via videoconferencing), (ii) outpatient telephone encounters, (iii) emergency department presentations, (iv) hospital admissions, (v) days admitted to hospital, and (vi) inpatient consultations. Healthcare utilization information was collected from local and regional electronic medical records.

#### Intervention

The group intervention administered in this study was designed for individuals with SSD based on CBT principles, and by extrapolating components from the most promising findings in the non-pharmacological treatment-related findings in the SSD literature. The novel group intervention drew on CBT principles from a well-established body of literature and applied theoretical tenets in order to meet the clinical needs of this unique patient population in a real-world setting [56, 57]. Based on our review of the literature on treatments for SSD as well as studies on SSD and its comorbidities, we developed an approach that prioritized symptom reduction, skill improvement, and empowerment, which is very much consistent with the findings from Hijne et al.’s concept mapping study of factors influencing goal attainment in patients with SSD [58]. The resulting group integrated a standard CBT approach with aspects from several third wave approaches including acceptance and commitment therapy and dialectical behavior therapy. The intervention consisted of six weekly sessions which were each two hours in length. Homework was assigned at the end of each session and collaboratively reviewed at the beginning of the subsequent session. The first session provided psychoeducation about somatic symptoms and introduced the CBT theory and framework. The second session focused on cognitive restructuring to help participants learn to modify dysfunctional beliefs and increase awareness of emotions pertaining to somatic symptoms. The third session focused on applying relaxation and mindfulness techniques to reduce distress associated with somatic symptoms. The fourth session covered behavioral principles, such as time-based pacing of activities, sleep hygiene, and the importance of diet and exercise on physical health and wellness. The fifth session integrated concepts from acceptance and commitment therapy [57], addressing how experiential avoidance perpetuates physical and emotional difficulties in the long run, and the alternative of accepting the current circumstances and striving to live in accordance with one’s values. The final session focused on communication skills, drawing on concepts from dialectical behavior therapy [59].

### Study clinicians

This pilot study was conducted in a real-world clinical setting to meet the needs of referred psychiatric patients to a large outpatient mental health facility. Group facilitators included two fellows and an advanced graduate student in clinical and neuropsychology, all of whom were experienced in providing CBT. The fellows performed the initial screening evaluations and clinical interviews. The fellows and graduate student were supervised by a licensed Ph.D. level clinical psychologist and board-certified neuropsychologist.

## Results

### Profile of individuals presenting for treatment for SSD

In participants with a PAI somatization scale total score of *T* ≥ 70; (*n* = 13), the number of comorbid psychiatric conditions experienced ranged from 0 to 4, with 62% of participants reporting at least one comorbidity (*n =* 8) and approximately 46% reporting two or more psychiatric comorbidities. The most prevalent comorbidities observed in the somatic sample were major depressive disorder/persistent depressive disorder (31%, *n =* 4) and generalized anxiety disorder (31%, *n =* 4). Baseline somatic symptom severity (PHQ-15) demonstrated significant moderate to strong positive correlations with pre-group levels of depression (*r* = .67, *p* < .05; PHQ-9), anxiety (*r* = .77, *p* < .01; GAD-7), perceived disability related to pain (*r* = .61, *p* < .05; PDI), and perceived stress (*r* = .74, *p* < .01; PSS-4) (Table 2).

**Table 2:** Bivariate correlations of somatic symptom severity (PHQ-15), psychological, and health outcome measures at baseline

	1	2	3	4	5	6	7
1. Symptom Severity	-	0.77**	0.67*	0.74**	0.26	0.17	0.26
2. Anxiety	0.77**	-	0.75**	0.84**	− 0.08	− 0.07	0.08
3. Depression	0.67*	0.75**	-	0.92**	0.23	0.21	0.35
4. Perceived Stress	0.74**	0.84**	0.92**	-	0.18	0.12	0.37
5. Pain Disability	0.26	− 0.08	0.23	0.18	-	0.53	0.52
6. Personal Control	0.17	− 0.07	0.21	0.12	0.53	-	0.73**
7. Treatment Control	0.26	0.08	0.35	0.37	0.52	0.73**	-

*Note*: ** is significant at the 0.01 level. * is significant at the 0.05 level

Healthcare utilization behaviours were most notable for outpatient visits (*M* = 8.64, *SD* = 4.34), in which values ranged from 2 to 18 visits over the preceding 6 months. Although records indicated that approximately 31% of participants visited the ER in the 6 months prior to the group; the prevalence of the remainder of inpatient-related activities (i.e., hospital admissions, days admitted to hospital, inpatient consults) in the sample was observed to be considerably smaller, comprising approximately 15% of participants (*n* = 2) (Table 3).

**Table 3:** Pre-group means of healthcare utilization metrics

	Mean	SD
Outpatient Visits	8.64	4.34
Outpatient Telephone Visits	1.09	1.22
Hospital Admissions	0.27	0.65
Days Admitted to Hospital	4.82	14.11
Inpatient Consults	0.64	1.80
ER Visits	0.45	0.69

### Treatment completers versus non-completers

Independent samples t-tests were conducted comparing baseline psychological and health functioning of individuals who completed the group cycle (i.e., treatment completers, *n* = 11) and those who opted to discontinue prematurely (i.e., treatment non-completers, *n* = 2). Non-completers (*M* = 16.50, *SD* = 3.54) reported significantly higher baseline levels of anxiety (GAD-7) than treatment completers (*M =* 8.55, *SD* = 4.70), *t*(11) = -2.25, *p* = .05. Further, non-completers (*M* = 90.00, *SD* = 0) reported significantly higher baseline levels of health concerns (PAI) than treatment completers (*M* = 85.00, *SD* = 6.87), *t*(11) = − 2.41, *p* = .04. No other baseline psychological or health outcome measures demonstrated a significant difference between the two cohorts; however, notable trends included higher levels of conversion (PAI), somatization (PAI), somatic symptom severity (PHQ-15), depression (PHQ-9), and perceived stress (PSS-4) in non-completers. Treatment completers reported higher perceived disability related to pain (PDI), personal control (IPQ-R), and treatment control (IPQ-R) (Table 4).

**Table 4:** Results of independent samples t-tests examining the differences in the profile of treatment completers versus non-completers at baseline

	Completers		Non-Completers		*t*(11)	*p*
	Mean	SD	Mean	SD		
SOM Total	84.27	9.51	94.00	0	-1.40	0.19
Conversion (PAI)	77.45	17.69	84.00	4.24	− 0.50	0.63
Somatization (PAI)	76.55	10.18	91.50	3.54	-1.99	0.07
Health Concerns (PAI)	85.00	6.87	90.00	0	-2.41	0.04*
Somatic Symptom Severity	14.73	5.85	19.00	2.83	− 0.99	0.35
Anxiety	8.55	4.70	16.50	3.54	-2.25	0.05*
Depression	14.27	7.23	22.00	2.83	-1.45	0.18
Perceived Stress	9.36	3.56	14.50	0.71	-1.97	0.08
Pain Disability	47.14	11.20	39.50	27.58	0.39	0.76
Personal Control	18.64	3.61	18.50	0.71	0.05	0.96
Treatment Control	14.09	3.30	14.00	1.41	0.04	0.97

*Note.* Equal variances not assumed for the following variables: SOM-H (df = 10); Pain Disability (df = 1.06). * is significant at the 0.05 level

### Symptom and healthcare utilization changes

Paired samples t-tests did not reveal significant differences pre- to post-group in participants’ experiences of somatic symptom severity and/or other associated psychiatric symptoms (Table 5). Of note, we observed reductions in the mean scores for somatic symptom severity (PHQ-15), depressive symptomatology (PHQ-9), anxiety (GAD-7), perceived stress (PSS-4) and perceived disability related to pain (PDI). The change in depressive symptomatology pre- to post-group yielded a small effect size (*d* = 0.30). The findings also demonstrated trends towards higher levels of personal control (IPQ-R) post-group. Some evidence for improvements in pain management self-efficacy were also observed; however, data on this measure was only available for a subset of participants (*n* = 8).

**Table 5:** Results of paired samples t-tests examining the effect of a CBT-Pilot group for SSD on psychological and health outcome measures

	Pre-Group		Post-Group		*t*(10)	*p*	Cohen’s *d*	Descriptor
	Mean	SD	Mean	SD				
Somatic Symptom Severity	14.73	5.85	14.55	4.87	0.24	0.81	0.03	Decrease
Anxiety	8.55	4.70	7.82	4.85	0.67	0.52	0.15	Decrease
Depression	14.27	7.23	12.36	7.49	1.69	0.12	0.30*	Decrease
Perceived Stress	9.36	3.56	8.82	4.00	0.60	0.56	0.14	Decrease
Pain Disability	47.14	11.20	45.27	10.08	0.74	0.48	0.17	Decrease
Personal Control	19.00	3.59	19.40	2.95	− 0.32	0.75	− 0.12	Increase
Treatment Control	14.00	3.46	13.80	4.47	0.18	0.86	0.05	Decrease
Pain Self-Efficacy	21.13	7.26	23.13	8.53	− 0.72	0.49	− 0.25	Increase

*Note.* Sample size was n = 11 for all pre- and post-group outcome variables with the exception of the following: personal control (n = 10); treatment control (n = 10); pain self-efficacy (n = 8). * = small effect size

In a series of paired samples t-tests, downward trends were seen in the following mean healthcare utilization outcome metrics: outpatient visits, hospital admissions, days admitted to hospital, inpatient consults, and ER visits for group participants (Table 6). There was no change in the number of outpatient telephone visits from pre to post-group. Notably, small effect sizes were observed for hospital admission (*d* = 0.36), days admitted to hospital (*d* = 0.47), and inpatient consults (*d* = 0.42). There was a total of three hospital admissions for two participants in the 6-month before the group, and one participant was admitted to hospital once in the 6-month following the group. The total number of days admitted to hospital across all participants was 53 days pre-group and one day post group. Pre-group there were a total of 7 inpatient consultations and post-group there was one.

**Table 6:** Results of Paired Samples t-Tests Examining the Effect of a CBT-Pilot Group for SSD on Healthcare Utilization Metrics

	Pre-Group		Post-Group		*t*(10)	*p*	Cohen’s *d*	Trend
	Mean	SD	Mean	SD				
Outpatient Visits	8.64	4.34	8.45	6.98	0.10	0.92	0.03	Decrease
Outpatient Telephone Visits	1.09	1.22	1.09	2.39	0	1.00	0	Null
Hospital Admissions	0.27	0.65	0.09	0.30	0.80	0.44	0.36*	Decrease
Days Admitted to Hospital	4.82	14.11	0.09	0.30	1.11	0.29	0.47*	Decrease
Inpatient Consults	0.64	1.80	0.09	0.30	0.97	0.36	0.42*	Decrease
ER Visits	0.45	0.69	0.36	0.92	0.29	0.78	0.11	Decrease

*Note*: * = small effect size

## Discussion

Findings from this pilot study demonstrate promising trends, including the reduction of SSD symptoms and related symptomatology (i.e., depressive symptoms, anxiety, stress, and perceived disability) associated with participation in a six-session CBT-based intervention group. The current study indicates that group participation may also have implications for reductions in healthcare utilization among individuals with SSD. Though many results in this study did not reach statistical significance due to the small sample size, we propose that the degree of reduction in symptoms and healthcare utilization behaviours observed in the current study are suggestive of clinically meaningful benefit. Indeed, the trends toward declines in hospitalizations and days admitted to hospital among our pilot group participants suggest that participation in group CBT may help to prevent the degree to which individuals with SSD utilize costly healthcare services. Specifically, there were a total of 3 hospitalizations and 53 days admitted to hospital across participants in the six months prior to the group. In contrast, only one participant was admitted to hospital for only one day post-group. Given that the average hospital admission in Ontario costs nearly $7000 [60], this intervention may have contributed to significant savings in health care expenditure. The reductions in health care utilization have considerable implications for individual well-being as well as efficiency of health care spending.

While previous research on group CBT for SSD typically included 8 to 13 sessions [21, 34–37], these results suggest that a 6-session group may be sufficient treatment duration to reduce symptom severity and healthcare utilization. The abbreviated nature of this group may have significant implications for reductions in the considerable healthcare costs incurred in SSD. Further, the decrease in symptoms of depression and anxiety over the course of the CBT group were notable, as many of these individuals had been referred to the CBT group after other mental health treatments had proven to be ineffective. Current findings also revealed a pattern of increased perception of control and pain management self-efficacy over the course of the group. Specifically, individuals involved in the group appeared to show increased perceived control over their symptoms, including pain, during the course of the group. This is important given that higher self-efficacy has been shown to be negatively associated with the likelihood of having a functional somatic disorder [61]. Furthermore, researchers have found that higher self-efficacy is associated with lower symptom severity in similar disorders such as fibromyalgia, chronic pain, and chronic fatigue [62, 63]. This suggests that higher self-efficacy in those with SSD may help to reduce the severity of somatic symptoms. These findings are consistent with the overarching aims of our pilot study (i.e., mitigate symptoms, reduce healthcare utilization) and are in keeping with the CBT perspective (i.e., modify unhelpful thoughts, behavioural activation, improve understanding and ability to pace activities). In sum, the current study demonstrates the value of abbreviated group CBT to reduce somatic symptoms, affective symptoms, and healthcare utilization, while contributing to gains in self-efficacy—all of which are key concerns regarding treatment of individuals with somatic symptom disorders [10, 21, 22, 61].

There is a high rate of treatment drop-out and reduced engagement among those with somatic complaints [64]; thus, it is crucial that a better understanding of factors that increase the likelihood of patient attrition during the treatment of SSD is developed. Relatedly, the current findings showed individuals with higher SSD severity and lower perceived control were more likely to drop out than other group members. This is consistent with a study in a chronic pain sample finding that low perceived control was associated with increased early and late dropout rates [65]. In addition, individuals with higher levels of anxiety and health concerns tended to not complete the treatment. These are important findings and may assist clinicians in navigating work with individuals who present with more severe symptoms, as they may be more hesitant or more challenging to engage in treatment. An alternative approach centered on rapport building or individualized treatment may be better suited for these individuals. Indeed, the findings suggest that building personal control, instilling self-efficacy, and reducing anxiety may be crucial in early sessions.

### Limitations

This pilot study was conducted in an outpatient clinic in an effort to evaluate the clinical utility of including a CBT group for SSD among the clinical services that are offered. As a result, it is limited by a small sample size and lack of a control group. The sample was heterogeneous and included individuals with a variety of comorbidities, which is representative of the typical SSD population. There were many participant factors that were not controlled, such as medications and participation in other interventions. The group intervention was CBT-focused, with elements of third-wave CBT methods, including acceptance and commitment therapy and dialectical behavior therapy. While third-wave CBT approaches such as mindfulness and social skills training are conceptually relevant to the overarching treatment goals of individuals with SSD [58], the inclusion of such interventions may not lend to direct comparisons to other classic CBT groups (i.e., those which do not emphasize third-wave elements) in the research literature. Though the data analysis set only included individuals with clinically significant symptoms of SSD, a few individuals with subclinical somatic symptoms (not included in the data analysis) participated in the group, which may have reduced group cohesion. Group process measures such as cohesion were not included in the current study; thus, authors are unable speak to the degree that group process factors influenced participant adherence and outcomes based on the available data. Lastly, one of the major strengths of this study is the comparison of healthcare utilization before and after the group; however, this did not capture all forms of healthcare utilization in that information regarding visits with practitioners external to the healthcare system was not obtained.

### Future directions

Given the inherent challenges associated with treating this disorder and the resultant limited resources available to treat individuals with SSD, we suggest that group CBT targeted for somatic symptoms should be considered in addition to treatment as usual (e.g., individual psychotherapy; psychopharmacological intervention). This pilot study suggests that CBT groups for SSD in outpatient settings holds promise, and justifies resources being allotted into further developing and extending the intervention program. Nonetheless, group CBT interventions for SSD continue to warrant further investigation to improve our understanding of best and most effective practices. Future research should implement a randomized controlled trial design with more stringent experimental controls and a larger sample size in order to more effectively evaluate the efficacy of the intervention in reducing symptoms and healthcare utilization. Authors would also recommend that future studies use a standard CBT approach for treating SSD, particularly if the intervention is brief and one of the goals is to generalize the results to the research literature. Additional elements could be added in the future, and researchers could examine whether adding elements from other modalities (e.g., mindfulness-based and social skills-training interventions) benefit participant outcomes. Given the wide variability seen in healthcare utilization among those with SSD, future studies could investigate factors that contribute to healthcare utilization in this population and whether it would be prudent to target the intervention to those with higher healthcare utilization rates. Comprehensive information about healthcare usage would allow researchers to have a more fulsome picture of healthcare utilization, as many individuals with SSD access care across several disciplines. It will be important for future studies to compare the efficacy of abbreviated group treatments with their more traditional, longer counterparts and with individual therapy. A cost-benefit analysis would help to evaluate the net impact of a shorter, less expensive intervention that can be delivered to more people, considering the relatively high prevalence of SSD, high cost of healthcare utilization in this population, and current lack of access to treatment. Finally, more research is also needed to compare with other psychotherapeutic approaches for treating SSD as well as transdiagnostic factors contributing to somatization.

## Conclusions

The current study represents one of the few studies to date measuring both self-reported symptoms and healthcare utilization during a group CBT intervention for SSD. Symptom severity and healthcare usage are each important in SSD, given the complexity of the population. Findings of the study demonstrated a decrease in somatic and affective symptoms over the course of the group and a concomitant trend toward lower healthcare utilization in individuals who completed the treatment groups. Finally, the study revealed insights into the difficult nature of intervention in this population by highlighting that those individuals with lower perceived control and self-efficacy and higher overall symptom presentation were more likely to drop out of treatment. Given the significant burden of SSD on the individual (e.g., psychiatric comorbidities, decreased quality of life) and society (i.e., inundated healthcare systems), moving towards a better collective understanding of SSD may hold significant implications toward better intervention approaches for individuals with somatic symptom disorders.

## Data Availability

andmaterials. The dataset generated and analyzed during the current study is not currently publicly available as release of data was not included as part of the informed consent process and was not approved by the ethics board, but it may be available from the corresponding author on reasonable request and with ethics board approval.

## List of abbreviations

CBT: Cognitive behavioural therapy
GAD-7: Generalized Anxiety Disorder 7
IPQ-R: Revised Illness Perception Questionnaire
PAI: Personality Assessment Inventory
PCI: Psychiatric consultation intervention
PDI: Pain Disability Index
PHQ-9: Patient Health Questionnaire 9
PHQ-15: Patient Health Questionnaire 15
PSEQ: Pain Self-Efficacy Questionnaire
PSS-4: Perceived Stress Scale 4
SOM: Somatic Complaints Scale of PAI (Table 1)
SSD: Somatic symptom disorder
